# Factors associated with low prevalence of Fuchs' uveitis syndrome in Japan

**DOI:** 10.3389/fmed.2022.999804

**Published:** 2022-09-30

**Authors:** Yu Yoneda, Yoshihiko Usui, Rie Tanaka, Keitaro Hase, Kenichi Namba, Koju Kamoi, Hiroshi Takase, Masaki Takeuchi, Wataru Matsumiya, Sentaro Kusuhara, Atsunobu Takeda, Nobuyo Yawata, Ryoji Yanai, Tomona Hiyama, Yosuke Harada, Noriyasu Hashida, Kazuichi Maruyama, Kei Nakai, Ryo Taguchi, Toshikatsu Kaburaki, Nobuhisa Mizuki, Hiroshi Goto, Yujiro Fujino, Masaru Takeuchi

**Affiliations:** ^1^Department of Ophthalmology, National Defense Medical College, Tokorozawa, Japan; ^2^Department of Ophthalmology, Tokyo Medical University, Tokyo, Japan; ^3^Department of Ophthalmology, The University of Tokyo Hospital, Tokyo, Japan; ^4^Department of Ophthalmology, Faculty of Medicine and Graduate School of Medicine, Hokkaido University, Sapporo, Japan; ^5^Department of Ophthalmology and Visual Science, Graduate School of Medical and Dental Sciences, Tokyo Medical and Dental University, Tokyo, Japan; ^6^Department of Ophthalmology and Visual Science, Yokohama City University Graduate School of Medicine, Yokohama, Japan; ^7^Division of Ophthalmology, Department of Surgery, Kobe University Graduate School of Medicine, Kobe, Japan; ^8^Department of Ophthalmology, Kyushu University Graduate School of Medicine, Fukuoka, Japan; ^9^Department of Ophthalmology, Yamaguchi University Graduate School of Medicine, Yamaguchi, Japan; ^10^Department of Ophthalmology and Visual Science, Graduate School of Biomedical Sciences, Hiroshima University, Hiroshima, Japan; ^11^Department of Ophthalmology, Osaka University Graduate School of Medicine, Osaka, Japan; ^12^Department of Ophthalmology, Yodogawa Christian Hospital, Osaka, Japan; ^13^Department of Ophthalmology, Saitama Medical Center, Jichi Medical University, Omiya, Japan; ^14^Department of Ophthalmology, Japan Community Health Care Organization Tokyo Shinjuku Medical Center, Tokyo, Japan

**Keywords:** non-infectious uveitis, Fuchs' uveitis syndrome, Fuchs' heterochromic iridocyclitis, uveitis, cataract

## Abstract

**Aim:**

To investigate the causes of low prevalence of Fuchs' uveitis syndrome (FUS) in Japan.

**Methods:**

Medical records of 160 patients diagnosed with FUS at 14 uveitis specialty facilities in Japan were reviewed retrospectively.

**Results:**

In 160 FUS patients, mean follow-up period before referral to our uveitis facilities was 31.6 ± 50.9 months. The most common reason for referral was idiopathic uveitis (61.9%), followed by cataract (25.0%), high intraocular pressure (IOP) including glaucoma (16.3%), and FUS (14.4%). Unilateral involvement was 96.9%. The most frequent ocular finding of FUS was anterior inflammation (91.9%), followed by stellate-shaped keratic precipitates (88.1%), cataract/pseudophakia (88.1%), diffuse iris atrophy (84.4%), vitreous opacity (62.5%), heterochromia (53.1%) and high IOP including glaucoma (36.3%). As treatments of these ocular findings, cataract surgery was performed in 52.5%, glaucoma surgery in 10.6%, and vitrectomy in 13.8%. Mean logMAR VA was 0.28 ± 0.59 at the initial visit, and decreased significantly to 0.04 ± 0.32 at the last visit. Proportions of FUS patients with BCVA <0.1 and 0.1 to <0.5 decreased, while that of ≥0.5 increased at the last visit compared with the initial visit.

**Conclusions:**

Ocular findings of FUS in Japanese FUS patients were consistent with the characteristic features. The low prevalence of FUS in Japan may be a result of being overlooked and misdiagnosed as mild idiopathic uveitis, cataract, and/or glaucoma.

## Introduction

Fuchs' uveitis syndrome (FUS), first described by Ernst Fuchs in 1906, manifests chronic, granulomatous, and typically unilateral mild anterior segment inflammation (1, 2). FUS is also referred to as Fuchs' heterochromic iridocyclitis (3). The exact etiology of FUS is unknown, but many studies suggested an infectious theory associated with an inflammatory process to rubella (4–6). FUS is a distinctive form of uveitis with variable clinical appearances, and the diagnosis is made by the coexistence of characteristic clinical features of FUS including little or no ciliary injection, unique stellate-shaped keratic precipitates, low-grade iridocyclitis, lack of synechiae, iris atrophy with or without heterochromia, elevated intraocular pressure (IOP), posterior subcapsular lens opacities, and vitreous cells and opacity (7–11). Most patients may remain asymptomatic for years after onset, and become aware of symptoms such as decreased visual acuity and/or blurred vision due to progression of cataracts and vitreous opacity. Conservative treatment consists of corticosteroid eye drops, but anti-inflammatory treatment does not alter the clinical course. The outcomes of surgery for cataract and vitreous opacity complicating FUS are satisfactory, and the visual outcome of FUS is favorable (12–14).

Epidemiological features of FUS patient population including prevalence, symptoms according to age, and rates of complications vary by geographic location (8, 11, 15–18). Although epidemiology of uveitis differs among countries, the prevalence of FUS tends to be higher in developed countries and lower in developing countries (6, 19). The prevalence of FUS in most developed countries range from 1 to 11%, while that in Japan is 0.5–0.7%, which is apparently lower (19–22). Heterochromia is one of the hallmarks of FUS, but this finding may be absent (23) and is often disregarded in races with dark brown or black iris (24). In addition, ocular findings characteristic of FUS do not always coexist at the same time; if frequencies of characteristic findings are low in a population, FUS may be overlooked or cannot be diagnosed. In this study, we investigated the frequencies of ocular findings characteristic of FUS in Japanese patients and the clinical history, and examined factors associated with the lower prevalence.

## Materials and methods

### Subjects

This was a retrospective, multicenter cohort study. Medical records of FUS patients who were followed at 14 uveitis specialty facilities in Japan (National Defense Medical College, Tokyo Medical University, University of Tokyo, Hokkaido University, Tokyo Medical and Dental University, Yokohama City University, Kobe University, Kyushu University, Yamaguchi University, Hiroshima University, Osaka University, Yodogawa Christian Hospital, Saitama Medical Center of Jichi Medical University, and Tokyo Shinjuku Medical Center) between April 2010 and March 2021 were reviewed retrospectively. The study was conducted according to the tenets of the Declaration of Helsinki and was approved by the ethics committee of the National Defense Medical College Hospital (institutional review board number: 4365) and institutional review board of each of the other facilities. Written informed consent was waived by the ethics committees due to the retrospective nature of the study, but consent by opt-out approach was obtained by posting the study protocol on hospital websites.

### Data collection

Diagnostic criteria of FUS were established based on typical clinical findings as described in the literature and in the classification criteria proposed by SUN working group (10, 11, 16, 25, 26). Accordingly, patients were diagnosed with FUS if they showed most (although not all) of the following clinical features: unilateral, chronic, low-grade anterior chamber inflammation, no or little ciliary injection, typical stellate-shaped keratic precipitates, diffuse iris atrophy, heterochromia, cataract, and various degrees of vitreous opacity. The following data were collected: detailed clinical history including personal details and previous ophthalmological and medical histories, the reasons for referral to our hospital, follow-up duration at the primary general ophthalmologists before being referred to our uveitis specialty facilities, best corrected visual acuity (BCVA) and IOP at initial and final visits, ocular findings, and complications. In bilateral cases, only data from the right eye were used. For differential diagnosis, laboratory test results including angiotensin converting enzyme test, syphilis serology using a treponemal test, and herpes virus antibodies; tuberculin skin test; fundus fluorescein angiography; and chest radiograph were referred. Exclusion criteria were positive serology for syphilis, evidence of sarcoidosis, and PCR test of aqueous specimen positive for herpes simplex virus, varicella zoster virus, or cytomegalovirus (10).

### Statistics

Statistical analysis was performed using JMP Pro ver. 15 (Business Unit of SAS, Cary, NC). Continuous variables were compared by paired *t*-test, and categorical variables were analyzed using Pearson χ^2^ test. *P*-values lower than 0.05 were considered statistically significant.

## Results

**Figure 1:**
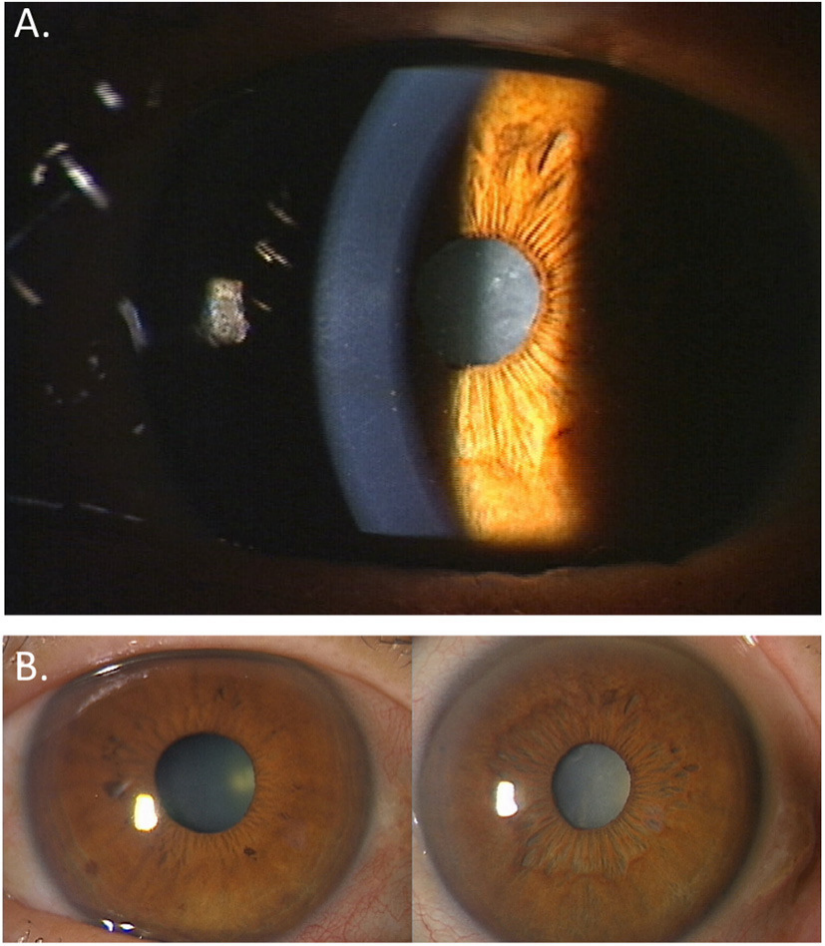
Ocular features in a patient with Fuchs' uveitis syndrome. **(A)** White, translucent, small- to medium-sized stellate-shaped keratic precipitates are observed. **(B)** Diffuse iris stromal atrophy and cataract are seen in the left eye compared to the normal right eye.

Clinical hallmarks for diagnosing FUS include stellate-shaped keratic precipitates (Figure 1A), anterior uveitis, IOP elevation, diffuse iris stromal atrophy (Figure 1B), cataract, and vitritis in the affected eye.

Table 1 shows the background data of 160 FUS patients enrolled in this study. Mean age at diagnosis was 48.3 ± 15.0 years (range, 16–89 years), and male to female ratio was 81:79, which was almost equal. Mean follow-up period at the primary general ophthalmologists before being referred to our uveitis specialty facilities was 31.6 ± 50.9 months (range, 0–240 months). One hundred and fifty-five out of 160 patients (96.9%) had unilateral FUS, while 5 patients (3.1%) had bilateral disease. Regarding subjective symptoms at initial presentation, decreased visual acuity was the most common symptom (41.3%), followed by blurred vision (38.1%) and floaters (7.5%). Hyperemia (2.5%) or irritation (1.9%) associated with ocular inflammation was rare, and 8.8% had no symptoms. The most common reason for referral was idiopathic uveitis (61.9%), followed by cataract (25.0%), high IOP including glaucoma (16.3%), and FUS (14.4%). Overall, 81.3% of the patients were referred for investigation of uveitis; however, approximately 40% were referred for diseases other than uveitis.

**Table 1:** Background of Fuchs' uveitis syndrome patients enrolled in this study.

	FUS^*^patients (*n* = 160)
**Age; years**	
Mean (SD)	48.3 (15.0)
Median (range)	46 (16–89)
Male/female	81/79
**Follow-up period at referral clinics; months**	
Mean (SD)	31.6 (50.9)
Median (range)	6 (0–240)
**Affected eye**	
Unilateral; no. of patients (%)	155 (96.9)
Bilateral; no. of patients (%)	5 (3.1)
**Subjective symptoms: no. of patients (%)** ^†^	
Decreased visual acuity	66 (41.3)
Blurred vision	61 (38.1)
Flatters	12 (7.5)
Hyperemia	4 (2.5)
irritation	3 (1.9)
No symptom	14 (8.8)
**Referral reasons; no. of patients (%)** ^†^	
Unidentified uveitis	99 (61.9)
Cataract	40 (25.0)
High IOP including glaucoma	26 (16.3)
FUS	23 (14.4)
Sarcoidosis	5 (3.1)
Herpetic iridocyclitis	3 (1.9)
Others	2 (1.3)

* FUS, Fuchs' uveitis syndrome;

† Including overlaps.

Ocular findings of FUS patients at initial presentation are shown in Table 2. Stellate-shaped keratic precipitates were observed in 141 patients (88.1%), and anterior ocular inflammation was found in 147 patients (91.9%), which was mostly mild with no cell infiltration of 2+ or more. While diffuse iris atrophy was also common, found in 135 patients (84.4%), heterochromia was seen in 85 patients (53.1%) and iris nodules in 40 patients (25%). In contrast, 108 patients (67.5%) had cataract and 33 patients (20.6%) had pseudophakia, totaling 141 patients (88.1%). Increased IOP including glaucoma was observed in 58 patients (36.3%), and vitreous opacity was found in 100 patients (62.5%). As treatments of these ocular findings, cataract surgery was performed in 52.5%, glaucoma surgery in 10.6%, and vitrectomy in 13.8%.

**Table 2:** Ocular findings of Fuchs' uveitis syndrome patients at the initial presentation and intraocular surgery performed thereafter.

	**Number (%)**
Stellate-shaped keratic precipitates	141 (88.1)
**Anterior chamber cells**	
0	13 (8.1)
1+	147 (91.9)
2+	0 (0)
Diffuse iris atrophy	135 (84.4)
Heterochromia	85 (53.1)
Iris nodule	40 (25)
Cataract	108 (67.5)
Pseudophakia	33 (20.6)
High IOP^*^/glaucoma	58 (36.3)
**Vitreous opacity**	
-	60 (37.5)
+	100 (62.5)
**Intraocular surgery**	
Cataract surgery	84 (52.5)
Glaucoma surgery	17 (10.6)
Vitrectomy	22 (13.8)

* Intraocular pressure.

Table 3 shows logMAR VA, BCVA, and IOP at the initial and the last visits. Mean logMAR VA was 0.28 ± 0.59 (range, −0.18 to 3) at the initial visit, and decreased significantly to 0.04 ± 0.32 (range, −0.10 to 2) at the last visit (*P* < 0.0001). The numbers of FUS patients with BCVA <0.1, 0.1 to <0.5, and ≥0.5 were, respectively, 20 (12.5%), 15 (9.4%), and 125 (78.1%) at the initial visit, and 5 (3.2%), 4 (2.6%), and 147 (94.2%) at the last visit. Proportions of FUS patients with BCVA <0.1 and 0.1 to <0.5 decreased, while that of ≥0.5 increased (*P* < 0.0001) at the last visit compared with the initial visit. Mean IOP also decreased significantly from 15.2 ± 7.0 (range, 6–51) at the initial visit to 13.4 ± 3.4 (range, 4–25) at the last visit (*P* = 0.0043), and the number of FUS patients with IOP higher than 20 mmHg decreased from 16 (10.0%) at the initial visit to 3 (1.9%) at the last visit (*P* = 0.0016).

**Table 3:** Visual acuity and intraocular pressure of Fuchs' uveitis syndrome patients at the initial and the last visit.

	**Initial visit (*n* = 160)**	**Last visit (*n* = 156)**	***P*-value**
LogMAR VA			<0.0001
Mean (SD)	0.28 (0.59)	0.04 (0.32)	
Median (range)	0.05 (−0.18 to 3)	−0.08 (−1.0 to 2)	
Best corrected visual acuity; no. of eyes (%)			<0.0001
<0.1	20 (12.5)	5 (3.2)	
0.1– <0.5	15 (9.4)	4 (2.6)	
0.5	125 (78.1)	147 (94.2)	
Intraocular pressure; mmHg			0.0043
Mean (SD)	15.2 (7.0)	13.4 (3.4)	
Median (range)	13.4 (6–51)	13.0 (4–25)	
Intraocular pressure >20 mmHg; no. of eyes (%)	16 (10.0)	3 (1.9)	0.0016

## Discussion

FUS is a type of non-granulomatous uveitis with latent onset and low-grade activity (27), usually without developing ocular pain, hyperemia or photophobia (18, 25). It is unlikely that FUS patients visit an ophthalmologist with complaints associated with anterior uveitis, and the diagnosis is frequently made during the course of routine ophthalmologic examinations for decreased visual acuity or blurred vision related to cataract, glaucoma, or vitreous opacity. Although, the involvement of rubella virus in the onset of FHC has been reported in recent years (4, 5, 28), no specific diagnostic test has been established to confirm the diagnosis of FUS, and the diagnosis is still based solely on clinical findings (10, 11). Therefore, FUS is often an overlooked cause of anterior uveitis, and misdiagnosis results in unnecessary tests and ineffective treatment.

Demographic and clinical features of FUS patients reported from various countries are presented in Table 4 (8, 11, 13, 16–19, 23, 29, 30). Ocular involvement was commonly unilateral, and bilateral involvement accounted for only 0–10.3%. There was no gender predilection, and most patients were between 20 and 40 years of age. Similarly, in our Japanese patients, only 5 patients were affected bilaterally (3.1%) and there was no gender difference. However, the mean age of 48.3 ± 15.0 years in our cohort was apparently higher. The difference may be due to racial difference, but one of the possible causes is the delay in the diagnosis of FUS in Japan, since most patients had been treated by the primary general ophthalmologists for a mean of ~30 months under a non-FUS diagnosis.

**Table 4:** Clinical findings in Fuchs' uveitis syndrome reported by previous studies.

	**USA** ^**24**^		**UK^18^**	**Sweden^29^**	**Netherlands^23^**	**Italy^17^**	**Spain^8^**	**Saudi Arabia^13^**	**Turkey^16^**	**China^11^**	**Mexico^30^**
	**White**	**Black**									
Number of patients	55	13	103	54	51	100	26	100	172	593	68
Mean age (range)	–	–	36.1 (8–71)	37.0 (19–57)	40 (17–71)	29.2 (8–64)	30.2 (9–50)	35.2 (10–70)	29.5 (10–75)	32.3 (8–70)	31 (5–80)
Gender (M: F)	–	–	50 : 53	21 : 33	30 : 21	51 : 49	12 : 14	55 : 45	75 : 97	46 : 54	37 : 31
Bilateral involvement	7	0	7.8	5.6	4	0.6	3.8	4.8	5.2	7.4	10.3
KP	90	100	83.8	100	88	95.6	100	90.2	96.7	91.7	80
Iris atrophy	48	61	89.3	100	100	86.8	14.8	100	88.4	100	50.7
Heterochromia	92	76	90.3	75.9	82	38.3	70.4	13.9	39.7	–	25.3
Cataract/surgical	75	23	80.2	92.6	82	63.5	77.8	85.6	69.1	72.3	69
phakia											
High IOP/glaucoma	11	38	26.2	11.1	22	20.1	14.8	27.6	14.8	37.4	30.7
VO	–	–	66.6	92.6	84	91.2	14.8	50	71.8	53.1	46.7

KP, keratic precipitates; IOP, intraocular pressure; VO, vitreous opacity; n.e., not evaluated.

Among the wide spectrum of clinical findings of FUS, a classic hallmark is keratic precipitates, which have been described as white, translucent, small- to medium-sized, stellate dots dispersed all over the corneal endothelium, which tend not to aggregate. The presence of these precipitates does not affect vision; as a result, patients are unaware of the condition (31). Similar to previous reports (Table 4), 88.1% of FUS patients in our cohort exhibited the characteristic keratic precipitates, although there were some individual differences in number, spread, and shape.

Changes in the iris are considered to be specific and representative signs of FUS, consisting mainly of anterior stromal atrophy, depigmentation of the iris stroma, and loss of the iris pigmented epithelium. Since iris atrophy as a result of these tissue changes is a critical factor in the diagnosis of FUS, the prevalence is high in the majority of previous reports as well as in the present study. On the other hand, heterochromia caused by depigmentation of the iris varies widely among ethnic groups, which is often underscored as a feature of FUS. In North America and Europe, heterochromia was observed in over 70% of FUS patients (18, 23, 24, 29), but in 13.9, 39.7, and 25.3% in Saudi Arabia (13), Turkey (16), and Mexico (30), respectively. Furthermore, heterochromia was present in only 24.7% of FUS patients in a north Indian population (32). The difference in prevalence is considered to be predominantly due to the color of the iris. Heterochromia is often subtle and may even be absent in FUS patients with dark or brown iris (24, 33). Although the prevalence of heterochromia in Japanese FUS patients has not been reported before, it was 58.1% in this study, which is slightly higher compared with previous reports of FUS patients with dark brown eyes.

The frequency of cataract/pseudophakia was higher than 70% among FUS patients in most of the previous reports (Table 4), and was also 88.1% in the current study. Recently, favorable visual outcome can be achieved with modern cataract surgical techniques and IOL implantation (12). In our cohort, 84 FUS patients with cataract underwent cataract surgery, and BCVA improved to 1.0 or better in 65 patients (75.6%) (data not shown).

High IOP/glaucoma is one of major features found in FUS, and the prevalence of high IOP/glaucoma was 36.2% in all our FUS patients. Only 10% of the patients had IOP higher than 20 mmHg at the initial visit, because most of them were treated with anti-glaucoma eye drops prescribed by the previous ophthalmologists. Including 10.6% of the patients undergoing glaucoma surgery after referral, these results are consistent with previous reports shown in Table 4.

Complication of vitreous opacity was observed in more than 50% of FUS patients in most of the previous reports, some exceeding 80% (17, 23, 29). In this study, vitreous opacity was found in 100 patients (62.5%), 13.8% of whom received vitrectomy. The severity of vitreous opacity varies, and evaluation is not definite. In addition, since vitreous opacity could not be evaluated in some of the 84 patients (52.5%) who underwent cataract surgery due to the progression of cataract, the real complication rate would be even higher. The characteristic observations in FUS are conventionally defined by anterior ocular findings, but it is known that inflammation is also seen in the posterior ocular segment (3, 33–35). Bouchenaki and Herbort reported that almost all patients with Fuchs uveitis had posterior ocular inflammation as indicated by vitreous opacity and hyperfluorescence of the optic disc revealed by fluorescein angiography (35). These authors remarked that the major cause of misdiagnosis and diagnostic delay of FUS is due to ophthalmologists not accepting vitreous involvement as an important feature of FUS, and focusing on anterior involvement because FUS is classically identified as an anterior uveitis (2).

Visual acuity outcome in FUS patients who received cataract surgery was reported to be favorable, with overall 85% achieving 20/40 or higher (12). In addition, Al-Mansour et al. (13) reported that only 10% of FUS patients showed decreased visual acuity during the follow-up period after being diagnosed with FUS, and visual acuity in most patients improved or did not change. In this study, the proportion of FUS patients with BCVA 0.5 or more was 78.1% at the initial visit to our facilities, and was further increased to 94.4% at the last visit. Only 3 patients had decreased BCVA (data not shown).

Since ocular inflammation in FUS is mild and there is no severe visual impairment until cataract and/or glaucoma progresses or vitreous opacity increases, FUS patients enrolled in this study had been treated at general ophthalmology clinics for several months or years. On the other hand, although the characteristic ocular findings of FUS were similar in Japanese patients and patients in other countries, the most common reason for referral to our facilities was undefined uveitis, followed by cataract and high IOP including glaucoma (Table 1). Thus, FUS was overlooked in most of the patients, and only 23 patients (14.4%) were diagnosed with FUS before being referred.

The limitations of our study were the retrospective design, diagnostic criteria of FUS determined by individual facilities, and medical records written by different observers, which may add bias to both the cross-sectional and longitudinal data in this study. In addition, it is undeniable that the vitreous was not observed carefully in our facilities, including the fact that fluorescein angiography was not performed in all cases.

## Conclusions

Japanese FUS patients in the present study showed characteristic ocular findings comparable with those reported in other countries, which may suggest that the prevalence of FUS, if examined appropriately by uveitis specialists, could be higher than that observed in previous Japanese studies (20–22). The causes of the low prevalence of FUS in Japan are speculated as follows: (1) patients were treated by general ophthalmologists because uveitis was mild and visual acuity was preserved; (2) FUS was not a well-recognized disease except by uveitis specialists, and most patients were followed for idiopathic uveitis, cataract, or high IOP including glaucoma; and (3) patients were not referred to uveitis specialists until their subjective symptoms were worsened by progression of cataract, glaucoma and/or vitreous opacity.

## Data availability statement

The original contributions presented in the study are included in the article/supplementary materials, further inquiries can be directed to the corresponding author/s.

## Ethics statement

The studies involving human participants were reviewed and approved by the Ethics Committee of the National Defense Medical College Hospital (institutional review board number: 4365). Written informed consent for participation was not required for this study in accordance with the national legislation and the institutional requirements. Written informed consent was not obtained from the individual(s) for the publication of any potentially identifiable images or data included in this article.

## Author contributions

MasakT has full access to all data in the study, takes responsibility for the integrity of the data, and accuracy of the data analysis. YY, YU, RiT, KH, KenN, KK, HT, AT, NY, RY, TH, YH, NH, KM, KeiN, RyT, TK, NM, HG, MasarT, and YF were collected the data. YY and MasakT designed the study and wrote the manuscript. All authors contributed to the article and approved the submitted version.

## Funding

The study was supported by grants from the Grants-in-Aid for Scientific Research C (M. Takeuchi, 20K09840) from the Japan Society for the Promotion of Science (number 24592689).

## Conflict of interest

The authors declare that the research was conducted in the absence of any commercial or financial relationships that could be construed as a potential conflict of interest.

## Publisher's note

All claims expressed in this article are solely those of the authors and do not necessarily represent those of their affiliated organizations, or those of the publisher, the editors and the reviewers. Any product that may be evaluated in this article, or claim that may be made by its manufacturer, is not guaranteed or endorsed by the publisher.
